# Challenges and Advances in Interventional Cardiology for Coronary Artery Disease Management

**DOI:** 10.3390/medicina60081323

**Published:** 2024-08-15

**Authors:** Leslie Marisol Lugo-Gavidia, Marco Antonio Alcocer-Gamba, Araceli Martinez-Cervantes

**Affiliations:** 1 Mexican Academic Consortium for Clinical Data Acquisition SC, Sinaloa 80230, Mexico; 2 Dobney Hypertension Centre, Medical School, University of Western Australia, Perth 6000, Australia; 3 Facultad de Medicina, Universidad Autónoma de Querétaro, Santiago de Querétaro 76180, Mexico; 4 Instituto de Corazón de Querétaro, Santiago de Querétaro 76180, Mexico; 5 Centro de Estudios Clínicos de Querétaro, Santiago de Querétaro 76180, Mexico

**Keywords:** coronary artery disease, percutaneous coronary intervention, endovascular

## Abstract

The development of percutaneous coronary intervention (PCI) has been one of the greatest advances in cardiology and has changed clinical practice for patients with coronary artery disease (CAD). Despite continuous improvements in operators’ experience, techniques, and the development of new-generation devices, significant challenges remain in improving the efficacy of PCI, including calcification, bifurcation, multivascular disease, stent restenosis, and stent thrombosis, among others. The present review aims to provide an overview of the current status of knowledge of endovascular revascularization in CAD, including relevant trials, therapeutic strategies, and new technologies addressing particular scenarios that can impact the prognosis of this vulnerable population.

## 1. Introduction

Cardiovascular diseases (CVDs), principally ischemic heart disease and stroke, are the leading cause of global mortality and a major contributor to disability [1]. Data from 2021 reported ~20.5 million deaths from cardiovascular conditions [2].

The introduction of percutaneous transluminal angioplasty (PTA) in 1977 marked one of the most important events for cardiology and transformed the management strategies for CVD. While percutaneous coronary interventions (PCIs) remain a major area of interventional cardiology, the use of endovascular procedures has extended to many other cardiovascular areas, including peripheral artery disease, valvular disease (e.g., transcatheter valvular replacement, MitraClip), atrial fibrillation (e.g., Ablation), hypertension (e.g., renal denervation), and congenital repair (e.g., an Amplatzer), among others.

**Figure 1:**
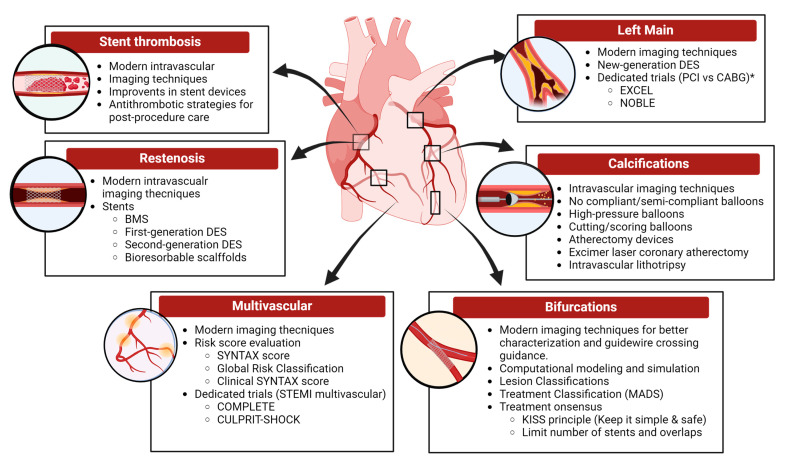
Graphical summary of challenges and complex circumstances encountered during coronary revascularization and advances in their management (including novel technologies, devices, and relevant trials). * Non-inferiority trials for PCI vs. CABG in left main using second-generation DES.

Coronary revascularization is the most important treatment strategy for coronary artery disease (CAD). Currently, international guidelines recommend coronary revascularization for patients with acute coronary syndromes (ACSs). It is also considered a valid strategy in stable coronary artery disease after appropriate evaluation [3]. Over the past decades, the development of new technologies and improvements in operator techniques have increased the efficacy and safety of endovascular procedures. Consequently, PCI has become the therapy of choice as it offers the advantage of minimal invasiveness and shorter hospitalization times; however, PCI poses multiple challenges. The aim of the present manuscript is to provide a brief yet comprehensive review of the significant challenges in the interventional management of CAD and the different efforts made over the years to overcome them (Figure 1).

## 2. Acute Coronary Syndrome

The spectrum of acute coronary syndrome (ACS) includes unstable angina, non-ST-segment myocardial infarction (NSTEMI), and ST-segment elevation myocardial infarction (STEMI), which are characterized by a sudden reduction in blood supply to the heart. Hence, emergent revascularization to restore blood flow is recommended by international guidelines. Time to treatment is vital for STEMI patients; primary percutaneous coronary intervention (pPCI) is the preferred reperfusion strategy when available in <120 min [4]. In some cases, pPCI is not an immediate option, in that case, fibrinolysis should be initiated within 12 h of symptom onset as part of a pharmaco-invasive strategy [5]. It is recommended to perform coronary angiography with PCI (if indicated) between 2 h and 24 h after successful fibrinolysis. In the event of fibrinolysis failure, emergency PCI should be performed as soon as possible [5].

## 3. STEMI Networks

Reperfusion is the cornerstone treatment in STEMI; however, it is time-sensitive, and achieving the recommended PCI-mediated reperfusion time (<120 min) has been a constant challenge for STEMI management, especially in emerging countries where healthcare is limited by the lack of resources, delayed patient presentation, and an absence of STEMI networks. The diagnosis of STEMI relies on clinical presentations, electrocardiogram findings, and biochemical evidence of myocardial injury. STEMI treatment should be coordinated by regional hospital networks, including emergency medical services, primary care, non-pPCI centers, and cardiology departments. Ideally, the STEMI network should include constant communication between non-capable PCI centers and 24/7 PCI hospitals, focusing on minimizing time-to-treatment [6,7]. In recent years, telemedicine programs have become promising alternatives for the prompt diagnosis and management of STEMI. Different programs have been developed to help implement STEMI networks across the world. The “Stent for Life” initiative was effective from 2008 to 2016 in 23 countries, mainly in Europe; it has been renamed to “Stent-Save a Life”. This initiative focuses on improving the implementation of STEMI guidelines by identifying local barriers and defining actions to increase access to pPCI [7]. Other international STEMI programs include the following: the Mission Lifeline program by the American Heart Association, the Service d’Aide Medicale Urgente (SAMU) network in France, and the Latin American Infarction Telemedicine Network (LATIN), which provides coverage for acute myocardial infarction to >100 million patients in four Latin American countries [6,7,8].

## 4. Stable Coronary Artery Disease

Approximately 20.1 million persons in the United States live with chronic coronary syndrome and 11.1 million Americans have chronic stable angina pectoris [9]. While revascularization in an ACS setting is recommended by all international guidelines, in patients with chronic coronary disease, the decision for revascularization requires a thorough examination. Diagnostic testing is based on pre-test probability; the non-invasive assessments include coronary computed tomography, stress cardiac evaluation, single-photon emission computed tomography (SPECT), positron emission tomography (PET), and cardiac magnetic resonance (C-MRI). The invasive assessment considers coronary angiography (ICA) as the gold standard [10]. The principal goal of myocardial revascularization in stable coronary artery disease is to improve symptoms and decrease the risk of adverse cardiovascular outcomes such as death, heart failure, and myocardial infarction. The 2019 ESC guidelines for diagnosing and managing chronic coronary syndromes recommend revascularization for patients who are symptomatic despite receiving optimal medical therapy (OMT) and/or for those in whom revascularization can improve prognosis [11]. Several efforts were made to compare OMT and PCI in this context. The COURAGE trial and the ORBITA trial showed neutral results but had small sample sizes [12]. New insights into the impact of invasive management in chronic CAD were provided with the ISCHEMIA (International Study of Comparative Health Effectiveness With Medical and Invasive Approaches) trial. The ISCHEMIA trial randomized patients with chronic coronary disease and moderate or severe ischemia to undergo revascularization (PCI or CABG) combined with medical therapy and lifestyle changes, or to an initial OTM strategy with lifestyle changes. Although this trial did not show a reduction in death or myocardial infarction, ISCHEMIA showed that an early invasive strategy resulted in better symptom relief and improved angina-related quality of life [13].

## 5. Role of Invasive Imaging in CAD Revascularization

For patients with stable CAD and inconclusive non-invasive tests, the severity and extent of atherosclerotic narrowing in a coronary artery can be assessed with modern intravascular imaging techniques. Brief descriptions of the new imaging techniques are provided below.

*Intravascular ultrasound (IVUS):* Intravascular ultrasound is useful for supplementing the two-dimensional angiography representation. IVUS offers a cross-sectional view of the lumen and helps to identify dissections, residual stenosis, in-stent stenosis, and other defects that may not be apparent in angiography. It also enables calcification quantification [14,15]. An initial lesion evaluation with IVUS can help to accurately assess the lumen length and diameter for appropriate stent sizing. Studies have suggested a minimal lumen area (MLA) of ≤4 mm^2^ as the cutoff for lesions requiring stent implantation. IVUS is limited to determining the functional significance of the lesion [14].

*Optical coherence tomography (OCT) imaging:* Similar to IVUS, coronary OCT provides a cross-sectional view of the vessel. It uses near-infrared light with a wavelength of ~1300 nm. It has a better resolution than IVUS (10–20 µm), allowing the distinction of the different vessel components (internal and external elastic laminae, intima, media, and adventitia). It can measure the thickness of calcified lesions [16]. It is more limited in depth when compared to IVUS (maximum tissue penetration of 1–2 mm) and requires blood clearance, which can limit its use in certain lesions (e.g., main left) [17,18].

*Fractional flow reserve (FFR):* It uses fluid dynamics principles to define a lesion-specific index reflecting the impact of coronary stenosis on myocardial perfusion. FFR is calculated as the maximum achievable blood flow in the presence of stenosis divided by the maximum flow without obstructing CAD. An FFR value of <0.8 has been used to define ischemia-producing stenosis that should be considered for revascularization [11,19].

IVUS and OCT are better tools for determining the anatomical extent of the disease, while FFR is used for assessing the hemodynamic significance of the stenosis [20].

These technologies provide valuable information during coronary interventions, not only in stable CAD but also in complex cases. They enable accurate assessment of coronary anatomy and early detection of complications that can impact short- and long-term outcomes (e.g., dissection, stent sub-expansion, lesion coverage, etc.). The use of these technologies has helped optimize interventions and can assist in selecting the best treatment strategy.

## 6. Restenosis

One of the biggest challenges of coronary interventions is restenosis. It is defined as ≥50% luminal obstruction on a vessel previously treated with angioplasty. In the early stages of PTA, balloon angioplasty showed high rates of symptomatic recurrence due to a high rate of restenosis (~30–40%), reflecting the natural healing process after the mechanical damage caused by angioplasty to the arterial vessel wall. Over the years, a series of different devices were developed to help prevent vascular recoil.

*Bare metal stents (BMSs):* The first devices were self-expanding BMSs; they showed better outcomes compared to balloon angioplasty alone [BENESTENT and STRESS trials] [21]; however, they presented a high rate of thrombosis intrastent (~30%). The introduction of balloon-expandable stents enabled high-pressure implantation, achieving a better expansion and apposition to the vascular wall. Additionally, the use of antithrombotic therapy helped to reduce the risk of thrombosis. Nevertheless, intrastent restenosis due to neointimal hyperplasia after the stent implantation continued to limit the long-term results

*Drug-eluting stents (DESs):* Stents coated with antiproliferative drugs were used to reduce cell proliferation inside the stent. The SIRIUS, RAVEL, and TAXUS trials evaluated first-generation DESs, showing a decrease in early restenosis and the need for new revascularizations [22,23]. However, a new problem emerged: long-term trials showed that the antiproliferative drug slowed endothelization, resulting in a longer exposure of the scaffold that could trigger platelet activation and increase the risk of late and very late stent thrombosis [24,25]. In the following years, second-generation DESs were developed by introducing more biocompatible polymers, new antiproliferative drugs (e.g., everolimus and zotarolimus), and better structures (thinner struts, more flexibility). These advances, in addition to longer and/or more potent antiplatelet therapy regimens, have decreased the rates of late stent thrombosis rates while maintaining good efficacy in preventing restenosis [26,27,28].

*Bioresorbable scaffolds (BVSs):* They were proposed as a way to prevent stent hypersensitivity. They are made from either metallic or polymeric scaffolds (e.g., magnesium, poly-L-lactic acid). They provide initial mechanical support and disappear after a period of time (9 months–3 years). The first devices showed a series of problems, including stent thrombosis and technical difficulties (e.g., low navigability, low radial force, thick struts, limited expansion range, propensity to fracture, and greater recoil) [29]. 

The Absorb everolimus-eluting poly(L-lactide) BVS (Abbott Vascular) was one of the first and most widely studied BVSs. Several trials demonstrated an increase in adverse event rates compared to DESs [ABSORB II 1-year follow-up primary MACCE 5% vs. 3%, *p* = 0.35; ABSORB III 3-year follow-up composite end-point, 13.4% and 10.4%, *p* = 0.06; 13.4% vs. 10.4%, *p* = 0.06, device thrombosis 2.3% vs. 0.7%; *p* = 0.01] [30,31,32], mostly attributed to mechanical properties and suboptimal techniques. The results of the ABSORB IV at the 5-year follow-up [31] were recently published. This study incorporated active training, mandatory target lesion dilatation, and the inclusion of ACS. The absolute target lesion failure (TLF) was 17.5% in the BVS group vs. 14.5%; this study also showed the risk period for increased events was limited to 3 years, with similar event rates afterward [31].

Other BVSs include magnesium-based stents (MgBRSs), which have thin strut thickness, high mechanical strength, and high elastic modulus but are prone to fractures [29]. Pooled outcomes of the BIOSOLVE-II and -III trials evaluating MgBRS implantation (Magmaris) at 3 years showed a TLF of 6.3% with no stent thrombosis [33]. Subsequently, the MAGSTEMI trial was developed to compare MgBRS vs. sirolimus-eluting stents in patients with STEMI. Higher rates of TLF were observed in the MgBRS group at 16.2% vs. 4 5.3%; *p* = 0.030; this difference was observed during the first year, with no further differences between 1 and 3 years [34].

Newer devices in this area are being developed to overcome some of the limitations mentioned above; however, they are still under investigation [35].

Intrastent restenosis can be classified as focal or diffuse according to its localization. Restenosis is a progressive disease and, in many cases, is asymptomatic. A proportion of patients will present with unstable angina or even as an ACS depending on its severity. In asymptomatic patients, treatment should be avoided if possible. Severe cases of stenosis (>75%) will require a reintervention. It can be treated with drug-eluting balloon angioplasty, atherectomy, cutting/scoring balloon, or the implantation of a new stent.

## 7. Stent Thrombosis

**Figure 2:**
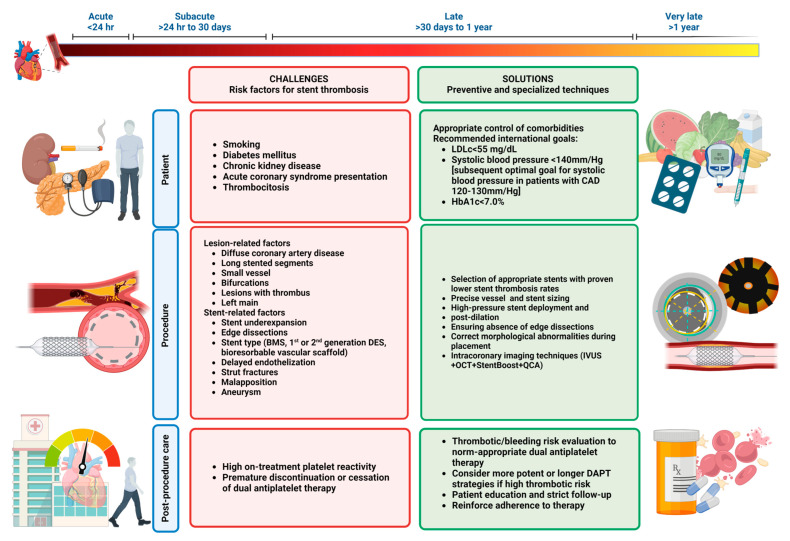
Factors related to stent thrombosis and preventive/treatment measurements.

Stent thrombosis (ST) is undoubtedly among the most feared complications of PCI as it has been associated with high mortality rates (10% to 25% at 30 days) and recurrence (~20% within 2 years). Fortunately, new improvements in devices, techniques, and antiplatelet therapies have helped to minimize its occurrence (~0.5% to 1%). The most susceptible period for ST is just after the stent implantation, the majority of ST occurs within 30 days of PCI; however, it can present later (0.2–0.6% per year), especially with devices like DESs and bioresorbable vascular scaffolds. Hence, it can be classified as early ST (acute—within 24 h after implantation and subacute—between 24 h and 30 days after implantation, late ST (30 days to one year after implantation), and very late (>1 year after implantation) (Figure 2) [36,37,38].

Multiple factors have been proposed to modify the risks of ST events, which can be grouped into the following categories: patient-related, procedure-related (lesion factors and stent factors), and post-procedural care factors (Figure 2).

Numerous clinical factors have been associated with worse prognosis in patients undergoing coronary stenting (clinical presentation, age, diabetes mellitus, chronic kidney disease, smoking status, peripheral artery disease, and reduced ventricular ejection fraction) [36,37,38].

Procedure-related factors involve the complexity of the lesion (such as diffuse coronary artery disease needing long stented segments, small vessels, bifurcations, lesions with thrombus, and left main involvement) and the features of the stent implanted [36,37,38]. The patient and lesion factors are hardly controllable by the interventionist before the PCI other than patient selection in elective procedures. Fortunately, new strategies and specialized techniques can be used during the intervention and secondary prevention management to minimize ST occurrence.

The appropriate selection of the stent is fundamental; bare metal stents typically present acute or subacute ST risks (Figure 2). The delayed integration of the stent into the vessel wall with the first-generation DES led to an increased risk of late ST. Second-generation DESs have reduced the ST risk by improving materials, resulting in thinner struts that are less toxic as well as more biocompatible devices; therefore, they should be preferred over older DESs. Despite the improvements in stent devices, some aspects of the intervention need to be taken into account. General recommendations include minimizing long stented sections, as well as the number of stents used; two-stent bifurcation techniques should be avoided [36,37,38].

New intracoronary imaging techniques such as IVUS and OCT are excellent tools for early detection of the underlying pathomechanism of ST [36,37,38]:

- Stent underexpansion: This is a common mechanism of ST; it can occur due to a stiff arterial segment or an undersized stent. It can be confirmed by IVUS or OCT and resolved with the use of a high-pressure balloon. If a high-calcium burden is detected, the use of rotational atherectomy can improve the stent deployment.
- Stent malposition: This occurs when there is a lack of contact between the struts and the intimal vascular wall of the vessel. It can be detected using imaging techniques (a space filled with blood is seen between the struts and vascular wall) and resolved with the stent post-dilatation.
- Stent edge dissections: This involves the mechanical disruption of the endothelium with blood extravasation into subendothelial planes [39]. Significant edge dissections (lumen narrowing < 4 mm^2^ or dissection angle > 60°) are associated with early ST. It can be detected with IVUS or OCT and treated with implantation of a second stent [14].
- Stent fracture: Tortuous or angled arteries, stent type, long stents, and/or overlapping stents are associated with the risk of stent fracture, which can promote ST. IVUS, OCT, StentBoost, and multislice CT scans can be used to diagnose. A thorough follow-up is required, and in some cases, re-stenting can be considered [40].
- Evaginations: Outward bulging of the luminal vessel contour between the stent struts. The use of intravascular imaging to ensure precise sizing can help prevent this issue.

Finally, post-procedural care and follow-up are essential to minimize ST occurrence. Dual antiplatelet therapy (DAPT) with a P2Y_12_ receptor antagonist in addition to aspirin is the cornerstone of ST prevention. Despite the compelling evidence of the importance of antithrombotic therapy for ACS management, the optimal duration and specific combination of therapy (e.g., potent P2Y_12_, such as prasugrel, ticagrelor) is still a matter of debate [36,37,38,41,42]. Current ESC guidelines for the management of acute coronary syndromes recommend a potent P2Y_12_ inhibitor (Prasugrel or Ticagrelor) plus aspirin for 12 as the standard regimen. However, different clinical scenarios require the establishment of alternative strategies (e.g., higher bleeding risk and the need for anticoagulation). In this context, several trials comparing different antithrombotic strategies have been published [5,41,43,44,45,46]. These trials can be divided into three groups: (a) shortening DAPT: these studies compared shorter DAPT duration (3 or 6 months) vs. standard or a prolonged (>12 months) regimen [EXCELLNT trial, RESET trial, OPTIMIZE trial, SECURITY trial, ISAR-SAFE trial, ITALIC trial, I-LOVE-IT 2 trial, IVUS XPL trial, NIPPON trial, REDUCE trial, MASTER DAPT trial]; (b) long-term DAPT trials: these studies evaluated the hypothesis that a longer DAPT duration (>12 months) was superior to the standard or a shorter regimen (6 months) [PRODIGY trial, DAPT trial, DES LATE trial, ARTIC-interruption, OPTIDUAL trial]; and (c) modified antithrombotic treatment: switching/DAPT de-escalation [TROPICAL-ACS, TOPIC trial, TALOS-AMI trial, HOST-REDUCE-POLYTECH-ACS trial], monotherapy with a P2Y12 inhibitor (after a short period of DAPT) [TWILIGHT trial, GLOBAL LEADERS trial, STOPDAPT-2 trial, TICO trial], dual therapy with low-dose anticoagulant [GEMINI-ACS-1] [5,41,42,43,44,45,46]. Although a detailed description of these investigations is not the goal of the present review, some considerations should be noted. The current evidence demonstrates a clear trade-off between the benefits obtained with longer or more potent strategies to prevent recurrent ischemic events and an increased risk of bleeding. Of note, both ischemic and bleeding events are known to have a negative impact on prognosis [41]. While scientific literature shows that shortening DAPT or de-escalating can be suitable alternatives for certain clinical scenarios to reduce the bleeding risk, they should not be employed as default strategies. Some of the limitations of these investigations include selection bias (low/medium risk patients, under-represented STEMI patients), non-inferiority design powered primarily by bleeding outcomes, and differences in the primary end-point definition/bleeding definitions [5,41]. Hence, 12-month DAPT continues to be the standard treatment in ACS. Treatment decisions should always be individualized, considering both thrombotic and bleeding risk [5,41,42,47].

## 8. PCI in Multivessel Disease

Multivascular disease is a common characteristic among patients with CAD. A proper evaluation of coronary anatomy and lesion features is fundamental to determining the best revascularization strategy. New imaging techniques can help to provide a better evaluation of the severity of the disease. Additionally, different risk scores have been developed to guide the therapeutic approach.

*SYNTAX score:* Evaluates functional and anatomical parameters to provide a comprehensive characterization of the coronary lesion. It includes features such as the segment affected in the coronary tree, diameter, bifurcations, the presence of calcium, and total occlusions, among others. It results in the following three risk categories: (1) low with a score < 22, (2) intermediate, i.e., between 23 to 32, and (3) high > 33. The score helps guide the optimal technique for revascularization, whether PCI for low-risk or CABG for high-risk [48,49].

*Global risk classification (GRC):* Combination of the EuroSCORE (European System for Cardiac Operative Risk Evaluation) and SYNTAX score, which identifies the following three risk categories: high (EuroSCORE 6 and SYNTAX score 26), intermediate (EuroSCORE 6 or SYNTAX score 26), and low (EuroSCORE 6 and SYNTAX score 26) [49].

*Clinical SYNTAX score:* Combines both angiographic and clinical characteristics for better prediction. Clinical characteristics include the following: the age, creatinine, and ejection fraction (ACEF) score [(age/ejection fraction) + 1, if for serum creatinine 2.0 mg/dL] [48,49].

Multivascular disease represents a particular challenge in STEMI patients as they often have additional lesions in locations separate from the culprit lesion that caused the acute event (~50%). The presence of multivessel disease confers a worse prognosis; however, the treatment of non-culprit lesions in STEMI patients has been controversial [50]. Multiple trials have evaluated preventive complete revascularization (PRAMI, CvLPRIT, DANAMI-3– PRIMULTI, and COMPARE-ACUTE). The most recent COMPLETE trial [complete versus culprit-only revascularization strategies to treat multivessel disease after early PCI for STEMI] demonstrated the superiority of complete revascularization (during the index hospitalization or soon after discharge) compared to culprit-lesion-only PCI in STEMI patients. This study showed a 32% lower risk of new, non-fatal myocardial infarction, and a 26% lower risk of a composite of death from cardiovascular causes or new myocardial infarction at the 3-year follow-up, without significant differences between the two groups for major bleeding or stroke [51].

Based on the results of these trials, current guidelines recommend complete revascularization of non-infarct-related arteries (IRAs), either during the index PCI procedure or within 45 days for STEMI patients [5]. In NSTE-ACS, complete revascularization with a functional invasive evaluation of non-IRA should be considered, preferably during the index procedure) [5]. Different considerations should apply when the ACS is complicated by cardiogenic shock. A high proportion of these patients will present multivascular disease (~80%), current guidelines recommend PCI to be restricted to IRA only during the index procedure. The CULPRIT-SHOCK trial demonstrated a reduction in all-cause death or renal replacement therapy at 30 days (RR 0.83, 95% CI, 0.71–0.96) with this approach [5].

## 9. Coronary Bifurcation Lesions

Coronary bifurcation lesions (CBLs) represent some of the biggest technical challenges in coronary interventions. CBLs account for approximately 15% to 20% of all cases requiring PCI. These interventions are associated with an increased risk of vascular occlusion, especially in the side branch, and also with a higher rate of restenosis [52]. The complexity depends on many factors, including the size of the vessels involved, the angle, whether the side branch ostium is affected, the carina morphology, and the characteristics and distribution of the atheromatous plaque [53]. CBLs can be classified based on the plaque distribution along the main (proximal or distal) and the side branch (Medina classification) [54]. Other CBL classifications have been proposed (e.g., Movahed Classification, Institut Cardiovasculaire Paris Sud (ICPS) classification) [54,55]; however, currently, the most commonly used is Medina’s classification due to its simplicity [54]. Additionally, the European Bifurcation Club proposed a treatment classification based on the position of the first stent called “MADS” (M: main vessel, A: across side branch, D: double stent implantation, S: side branch) [56]. The use of modern intravascular imaging provides a more accurate vessel evaluation (e.g., optimal projection angle with QCA, and calcium content with IVUS or OCT). OCT in particular can provide stent–vessel interaction analysis and visualization of guidewire recrossing through the stent side cells into the side branch [54]. New computational modeling and simulation provide insights into device performance, optimize stenting techniques, and facilitate preprocedural planning and training.

Several interventional techniques have been described for treating CBLs, including classic T stenting, modified T stenting, the crush technique, provisional stenting, the culotte technique, kissing stenting, crossover, and the proximal optimization technique (POT) [54,55]. A thorough revision of the particularities of these strategies is beyond the scope of this manuscript. Current guidelines and consensus statements recommend adhering to the KISS (keep it simple and safe) principle; this involves wiring both branches, limiting the number of stents and overlaps, and aiming for well-extended and well-apposed stents [54]. The provisional stenting strategy (also called the one-stent technique) is the first-line treatment for most CBLs except for those with complex anatomies and diffuse atherosclerotic involvement in the main vessel and side branch [56].

## 10. Left Main Coronary Artery

Left main coronary artery (LMCA) disease has a very high risk of mortality and morbidity as it provides almost 84% of the left ventricle blood supply (left dominant system) [57]. Current guidelines from the American College of Cardiology/American Heart Association and the European Society of Cardiology recommend revascularization for all patients with ≥50% stenosis of the LMCA regardless of symptomatic status or associated ischemic burden [58,59]. Intermediate LMCA stenosis is not infrequent, and angiographic evaluation can be challenging; in this context, IVUS is a useful tool to determine the severity of the lesion. The use of OCT is limited in ostial LMCA as it requires constant blood clearance. Given the complexity of LMCA, several clinical trials (the LEMANS trial, the SYNTAX-Left Main trial, and the PRECOMBAT trial) were conducted to evaluate the best revascularization strategy (PCI vs. CABG). Current guidelines recommend surgical revascularization for LMCA (class IA); PCI is considered a reasonable alternative in select patients with less complex anatomy (class IA for SYNTAX score 1–22 and class IIA for SYNTAX score 22–32). The findings from two recent trials comparing PCI to CABG, i.e., the EXCEL [60] and NOBLE trials [61], have special significance. These are the only two trials using second-generation DESs to test non-inferiority for PCI vs. CABG in LMCA. The EXCEL trial showed no significant difference between PCI and CABG at 5 years for the primary end-point (composite of death, stroke, or MI) with 22% in the PCI group and 19.2% in the CABG group (difference of 2.8%, 95% CI [−0.9, 6.5], *p* = 0.13) [60]. This was not the case in the NOBLE trial, where CABG was superior to PCI at the 5-year follow-up (28.4% MACCE for PCI vs. 19% for CABG with an HR of 1.58; 95% CI 1.24–2.01) [61]. The contradictory results can be explained by the difference in the endpoint definitions. The NOBLE trial used “non-procedural myocardial infarction” instead of “peri-procedural myocardial infarction” used by EXCEL and included repeat revascularization as part of its endpoint [61]. While it is clear that new-generation DESs and more modern intravascular imaging modalities have improved the treatment of LMCA with PCI, the best revascularization strategy for LMCA is still a matter of debate. The decision of the appropriate revascularization strategy should be made by a heart team considering the anatomic complexity and the clinical characteristics that predict an increased risk of adverse outcomes for PCI or CABG [59,62].

## 11. Calcified Coronary Lesions

Calcified coronary lesions are present in approximately one-third of CAD cases and increase with older age. They can be presented as flow-limiting stenoses or partially/non-obstructive lesions [63,64]. They can be detected via non-invasive methods (such as computed tomography) and quantified by the Agatston method. Severe calcification can be detected using fluoroscopy; this is defined as radiopacities observed without cardiac motion before contrast administration, involving both sides of the arterial wall in at least one location and a total length of calcium of at least 15 mm [64]. However, better accuracy is achieved via intravascular imaging as it can provide additional information to help fully characterize the lesion (e.g., superficial or deep wall calcium, calcified nodules, calcium extent including arc, length, and thickness) [15,16,64].

Calcified lesions are challenging to treat as the plaque becomes rigid and is difficult to cross and dilate using conventional devices. Additionally, calcified lesions present a risk of stent underexpansion and, consequently, increase the risk of MACCE. Interprocedural guidance with intravascular imaging has been helpful in identifying predictors of stent under expansion and developing better treatment strategies [64]. Calcium fractures improve lesion compliance and are essential for optimizing stent implantation. Several devices have been developed to assist in the treatment of calcified lesions.

*Balloons:* Balloon angioplasty can be used in less severe calcification or to prepare the lesion for further modification. The specialized balloons include semi-compliant and compliant balloons, high-pressure balloons, and cutting/scoring balloons. The purpose of balloon angioplasty is to create dissections in the media and disrupt the calcium to increase plaque elasticity and allow stent expansion, its primary risk is vessel perforation. Cutting balloons uses multiple microblades to create shallow incisions in the calcium plaque, allowing symmetrical stent expansion. Scoring balloons work similarly but deliver less trauma to the vessel wall [64].

*Atherectomy:* It is an important tool to disrupt the calcium plaque and prepare the lesion for stenting. Currently, there are different devices available. Rotational atherectomy (RA) (2a, level of evidence B-R 2021 ACC/AHA/SCAI recommendations for heavily calcified lesions) [58] uses a high-speed rotational device with a diamond-tipped device. RA requires continuous intracoronary infusion to reduce friction and heat; it should be performed by short ablations (<30 s) and avoid deceleration. Orbital atherectomy (OA) (2b, level of evidence B-NR 2021 ACC/AHA/SCAI recommendations for heavily calcified lesions) [58] enabled bidirectional atherectomy through a forward or backward motion and it creates elliptical orbits to induce calcium microfractures. Possible complications with both techniques include dissection, perforation, and transient conduction disorders. Finally, excimer laser coronary atherectomy (ELCA) uses ultraviolet light to modify the calcified plaque. It creates ablations, acoustic modification, and cavitation. Laser photochemical properties are useful for the ablation of softer luminal tissues; however, ELCA is limited to treating severe calcification [64]. 

*Intravascular lithotripsy (IVL):* It works with pulsatile sonic pressure waves to create superficial and deep, radial, and longitudinal micro- and macro-fractures. IVL has been shown to improve stent expansion [65]. It has good efficacy, especially in concentric calcified lesions, with a low periprocedural complication rate (<0.5%) [66].

All these technologies have led to improvements in the management of calcified lesions, however, there seems to be an underuse of dedicated devices due to the significant associated costs and the perceived higher procedural risk. Importantly, international societies have worked in expert consensus with technical recommendations to help mitigate the risk of complications.

## 12. Research Opportunities and Future Perspectives

Endovascular treatment for CAD has undergone a dramatic evolution, marked by an explosion of technological advancements over the decades. Yet, there are still opportunities to improve medical outcomes. Among the basic and translational science, new tests and biomarkers are being investigated to improve the evaluation of cardiovascular risks (e.g., platelet reactivity, high-sensitivity C-reactive protein, homocysteine, extracellular vesicles, pulse wave velocity, growth differentiation factor-15, fibrinogen, fractalkine, and genotyping) [67,68,69,70,71,72,73,74,75,76,77]. These tests may provide useful information and complement the assessment of selected patients. Furthermore, some of these markers have been linked to clinical outcomes, making them important for prognosis prediction.

Among new biological biomarkers, extracellular vesicles (EVs) play an important role in CAD, acting as links between thrombosis, endothelial dysfunction, and inflammation. Various studies have linked EVs not only with the presence of CAD but also with clinical and angiographical presentations. More importantly, EVs have been linked to worse clinical outcomes (higher EV levels were present during the index event in patients with a subsequent MACCE) [78,79,80,81,82]. Another important area is platelet function testing (PFT); numerous studies have shown variability in the response of antithrombotic therapy due to platelet reactivity. While PFT is not implemented routinary in clinical practice, high platelet reactivity on clopidogrel has been related to stent thrombosis [41,72,77,83]. The TROPICAL-ACS trial used PFT to guide the de-escalation of antiplatelet therapy from prasugrel to clopidogrel 2 weeks after ACS. The primary endpoint occurred in 7% of the guided de-escalation group and 9% of the control group (*p* for non-inferiority = 0.0004; hazard ratio [HR] 0.81 [95% CI 0.62–1.06], *p* for superiority = 0.12) [46]. Similar to PFT, genetic testing was also used to assess the cost-effectiveness of CYP2C19 genotype-guided treatment with antiplatelet drugs in patients with ST-segment-elevation myocardial infarction undergoing immediate PCI with stent implantation. This approach was evaluated in the optimization of treatment (POPular Genetics) trial to guide DAPT de-escalation, resulting in non-inferiority for thrombotic events and a lower bleeding incidence with this approach [84]. Biological molecules can help to improve guided therapies but are also being used to develop new targeted therapies (micro RNAs) and drug delivery systems.

The bioengineering field is developing new devices that incorporate new materials to make them more biocompatible. Also, they are incorporating biological coating (e.g., endothelial progenitor cells, Anti-CD34 coating) into the devices that can improve the healing process [85].

Finally, artificial intelligence (AI) and machine learning have been increasingly applied in the cardiovascular medicine field. AI has different applications, i.e., (a) it enables big data analytics to develop risk prediction models. (b) AI algorithms are being used in cardiovascular imaging processing.to quickly and accurately detect complex imaging features. AI can serve as a tool to identify new imaging biomarkers [86].

The use of state-of-the-art techniques and/or new biomarkers can help improve the performance of prediction models, identify patients prone to complications in earlier stages, and select more appropriate therapies. These are the first steps toward developing precision medicine, where more personalized and accurate strategies can be used.

## 13. Conclusions

Coronary artery disease remains the leading cause of death worldwide and is increasing rapidly. Coronary revascularization represents the standard of care for these patients. PCI has been an important breakthrough in cardiology, changing clinical practices for patients with CAD. Improvements in new-generation devices, imaging and procedural techniques, and post-procedural care factors have contributed to better outcomes; however, there are still significant challenges to overcome and opportunities for discovery and research in the endovascular field.
